# Entomopathogenic Fungi as Mortality Agents in Insect Populations: A Review

**DOI:** 10.1002/ece3.70666

**Published:** 2024-12-05

**Authors:** Robin Gielen, Kadri Ude, Ants Kaasik, Kadri Põldmaa, Tiit Teder, Toomas Tammaru

**Affiliations:** ^1^ Institute of Ecology and Earth Sciences University of Tartu Tartu Estonia; ^2^ Department of Ecology, Faculty of Environmental Sciences Czech University of Life Sciences Prague Prague Czech Republic

**Keywords:** Entomophthorales, Hypocreales, mycosis, population regulation, systematic review

## Abstract

Natural enemies play a key role in population dynamics of insects and exert significant selective pressures on various traits of these animals. Although there is a wealth of empirical and theoretical research on predators and parasitoids, the ecological role of pathogens (other than viruses) remains less understood. Entomopathogenic fungi (EPF), encompassing over 1000 known species from 11 phyla, have primarily been studied in the context of biocontrol in agroecosystems, while their role in natural ecosystems is poorly known. In this paper, we synthesize case studies reporting the prevalence of EPF infections in field populations of insects. We examine differences in this variable among major host taxa and those of the pathogens. From 79 case studies that met our selection criteria, we retrieved data on 122 species of fungi infecting 104 insect species. The meta‐analytic median prevalence of fungal infections was 8.2%; even if likely inflated by publication bias, this suggests that EPF‐induced mortality levels are lower than those attributable to predators and parasitoids. We found no substantial differences in fungal prevalence among major insect taxa and only a moderate difference among fungal orders, with Neozygitales showing the highest prevalence and Eurotiales the lowest. Our analysis revealed no significant differences in overall EPF prevalence between tropical and temperate studies, although different fungal taxa showed different geographical patterns. In temperate areas, there is some evidence of increasing infection prevalence toward the end of the growing season. Although quantitative data on the effect of EPF on insect populations are still scarce, evidence is consistent with the emerging generalization that insect populations commonly harbor species‐rich assemblages of pathogenic fungi, but infections rarely reach epidemic levels. Further studies on multi‐species assemblages of EPF associated with natural insect populations are needed to better understand the ecological role of fungal infections.

## Graphical abstract

We provide a quantitative review of studies reporting mortality attributable to entomopathogenic fungi in field populations of insects. Overall, data on this subject are surprisingly limited. Nevertheless, they support the conclusion that the assemblages of pathogenic fungi associated with insect populations are diverse but the prevalence mostly remains low.

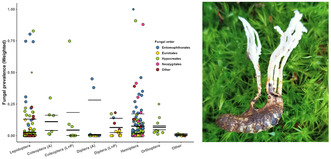

## 1 Introduction

High mortality rates constitute an inevitable counterpart to high fecundities characteristic of most insect species (Price et al. 2011). As a consequence, information on stage‐specific mortality is the key to understanding the ecology of insects, ranging from fundamental problems of life‐history evolution to applied questions of pest management (Cornell and Hawkins 1995; Peterson et al. 2009; Speight, Hunter, and Watt 2008). Almost invariably, a high share of juvenile insects succumb to various predators. Remmel, Davison, and Tammaru (2011) estimated that, in folivorous insects, daily mortality caused by vertebrate predators averages around 3%. However, values as high as 55% have been reported for late‐instar lepidopteran larvae (Nixon and Roland 2012). Similarly, invertebrate predators can reduce the expected lifespan of adult butterflies to just one day in temperate Europe (Sang and Teder 2011). Nevertheless, mortality rates are highly variable both in space and time. In this context, significant research efforts have focused on evaluating spatial variation in mortality patterns on the global scale. Invertebrate predators have been found to cause greater mortality in the tropics than at higher latitudes (Fricke et al. 2022; Roslin et al. 2017; Zvereva and Kozlov 2021), whereas mortality caused by vertebrate predators does not show such a trend (Roslin et al. 2017).

Apart from predators, in the second half of the 20th century, a substantial body of theoretical research focused on the role of (hymenopterous) parasitoids in the population dynamics of (primarily herbivorous) insects (e.g., Hassell 2000; Hawkins 1994; Hochberg and Ives 2000), with mixed support to the predictions from empirical studies (Mills 2010; Nixon and Roland 2012). In any case, the proportion of insects succumbing to parasitoids is substantial, with an estimated average of 13% (Peterson et al. 2009). However, much higher values are not uncommon (e.g., Teder and Tammaru 2003; Mills 2010; Nixon and Roland 2012).

Pathogens constitute another remarkably diverse functional group of insect mortality agents. Insect pathogens encompass beings as different as viruses, bacteria, protists, fungi, and nematodes (Roy and Cottrell 2013; Speight, Hunter, and Watt 2008; Vega and Kaya 2012). However, not least due to their cryptic nature (Bonsall 2004), mortality attributable to pathogens is often underestimated, and the ecological interactions involved are poorly known in general, especially in natural populations (Hawkins, Cornell, and Hochberg 1997; Meyling and Hajek 2010; Mora‐Aguilera, Cortez‐Madrigal, and Acevedo‐Sánchez 2017). Indeed, while the use of pathogens as biocontrol agents has stimulated a plethora of taxonomical studies and lab‐based research, ecological studies in this field are clearly lagging behind (Meyling and Hajek 2010; Mora‐Aguilera, Cortez‐Madrigal, and Acevedo‐Sánchez 2017; Vega et al. 2009).

Among pathogens, viruses are the most extensively studied ecologically, partly due to their potential as biocontrol agents (Vega and Kaya 2012). Insect viruses tend to be relatively host‐specific, often specializing at the level of insect order or family (Deka, Baruah, and Babu 2021). Their effect on natural insect populations is frequently density‐dependent (Il'inykh 2007), sometimes resulting in dramatic epidemics that lead to a rapid decline in insect populations (Bonsall 2004; Pell, Hannam, and Steinkraus 2010; Vega and Kaya 2012). In contrast, mortality attributable to bacteria is strongly linked to the environmental conditions and the specific pathogen strain involved (Jurat‐Fuentes and Jackson 2012).

Entomopathogenic fungi (EPF) in the strict sense are defined as those that have evolved physiological and behavioral traits allowing them to penetrate the cuticle of their living hosts (Boomsma et al. 2014; Roy and Cottrell 2013; St. Leger and Wang 2020). Unlike viral and bacterial pathogens, EPF can infect hosts without requiring ingestion of fungal propagules. Once the infection is established, the fungi propagate as single‐ or multicellular structures (protoplasts, blastospores, and/or hyphal bodies) within the host insect's body. Eventually, typical EPF kill their hosts and produce spores for transmission or form resting structures for persistence (such as sexual or asexual resting spores, chlamydospores, or by mummifying the hosts that persist in soil; Roy and Cottrell 2013). Entomopathogenic fungi s. str. appear to have evolved in 11 of the 18 fungal phyla recognized by Tedersoo et al. (2018): Rozellomycota (90 genera; Araújo and Hughes 2016; Solter, Becnel, and Oi 2012), Blastocladiomycota (2), Chytridiomycota (4), Basidiobolomycota (1), Entomophthoromycota (16), Zoopagomycota (4), Kickxellomycota (7), Mortierellomycota (2), Mucoromycota (2), Basidiomycota (3), and Ascomycota (14; only teleomorphs counted, Araújo and Hughes 2016; Gielen et al. 2021; Naranjo‐Ortiz and Gabaldón 2019; Samson, Evans, and Latge 2013).

Among the three fungal phyla comprising most entomopathogens, Rozellomycota encompasses intracellular parasites and pathogens with relatively narrow, species‐specific host ranges (Naranjo‐Ortiz and Gabaldón 2019; Tedersoo et al. 2018), known to infect insects from 12 orders (Araújo and Hughes 2016). These fungi are mainly microsporidian genera that rely on host ingestion of propagules for transmission (Araújo and Hughes 2016; Solter, Becnel, and Oi 2012). Infections, typically starting from the midgut, are chronic and often sublethal, resulting in lower fecundity of the host (Solter, Becnel, and Oi 2012). Fungi from Entomophthoromycota are highly specialized obligate entomopathogens, with known hosts from 10 insect orders (Naranjo‐Ortiz and Gabaldón 2019; Roy and Cottrell 2013). With some exceptions, these fungi primarily target adult insects (Araújo and Hughes 2016). Infection occurs via direct contact, after the attachment of the spore to the insect cuticle via a mucilaginous coat (Eilenberg, Bresciani, and Latgé 1986). These fungi are mainly biotrophic, feeding on their host only when it is alive.

Similarly, fungi from the phylum Ascomycota primarily infect insects through cuticle penetration, with the exception of *Ascosphaera*, which relies on oral ingestion. Ascomycetes are known to infect insects from 14 orders (Araújo and Hughes 2016) and exhibit varying degrees of host specificity, ranging from extreme specialists to broad generalists (Vega et al. 2012). Their feeding strategy is mainly hemibiotrophic, switching from a parasitic, biotrophic phase in the haemocoel to a saprotrophic phase, colonizing the host's body after death (Roy and Cottrell 2013; Samson, Evans, and Latge 2013). Successful infection by these fungi leads to the host's death (Samson, Evans, and Latge 2013), with the exception of ectoparasitic species from the order Labounbeniales (Haelewaters et al. 2021). The multitude of strategies of these fungi has spawned considerable confusion among mycologists, challenging the distinction between obligatory and facultative entomopathogens. As a result, fungal genera previously known to include saprotrophs or opportunists, such as *Fusarium* or *Penicillium*, have been neglected in the studies on entomopathogens (but see Gielen et al. 2022; Poitevin et al. 2018; Sharma and Marques 2018).

Although EPF have been the focus of diverse research efforts (Parsa, Ortiz, and Vega 2013; Pell, Hannam, and Steinkraus 2010; Vega 2018), our understanding of the role of these antagonists in the dynamics of insect populations remains limited, and we know virtually nothing about selective pressures that EPF impose on insect life histories (see, however, Rännbäck et al. 2015). There is no systematic understanding even of the most fundamental parameter, the mortality rate attributed to fungal entomopathogens in natural insect populations. Such information is mostly limited to case studies of commercially important pest species in agricultural settings (Hesketh et al. 2010; Roy and Cottrell 2013). Although EPF are considered to possess the ability to regulate insect populations (Pell, Hannam, and Steinkraus 2010; Steinkraus 2007), there is little evidence of density‐dependent mortality in natural settings (see, however Abney et al. 2008; Steinkraus 2007; Townsend, Glare, and Willoughby 1995). Even less is known about the diversity of fungal pathogen assemblages associated with particular insect populations (but see Gielen et al. 2021, Gielen, Põldmaa, and Tammaru 2022, Gielen et al. 2022, Gielen et al. 2023; Poitevin et al. 2018), as well as the spatial and phenological dynamics of the interactions between insect populations and their pathogens.

In this paper, we present a quantitative review of case studies documenting mortality rates attributed to EPF in field populations of insects. We focus on the overall distribution of mortality rates, as well as examine the differences in this parameter among major host taxa, and the taxa of the pathogens. As for more specific questions, we evaluate latitudinal clines in mortality caused by pathogenic fungi, and phenological patterns of EPF‐caused infections. Finally, we discuss the key questions that future research on the ecology of the insect–fungus interactions should prioritize.

## 2 Material and Methods

To analyze the prevalence of EPF in insect populations under field conditions, we made use of previously published empirical case studies in which insect sampling had been performed in the field, and the presence of fungal infection was documented at the level of insect individuals. In these studies, infected insects were typically found dead, showing visible signs of fungal growth on their bodies. This approach leaves infections by Rozellomycota undetected, as these pathogens do not leave such signs (Solter, Becnel, and Oi 2012), and consequently, infections by respective EPF thus do not appear in our database. As our focus was on pathogen‐induced mortality, we also did not include observations on Laboulbeniales, as these ectoparasitic fungi typically do not kill their hosts (Haelewaters et al. 2021). Additionally, we excluded studies examining EPF infections in social insects due to the unique ecological characteristics of these hosts.

Primary research papers (case studies) were collected through systematic searches in major literature databases (Google Scholar, Web of Science). Various combinations of search terms such as “entomopathogenic fungi”, “insect fungal disease”, and “insect + fungus + mortality” were used in the queries. Additionally, reference lists of relevant papers were examined. Source papers identified as “promising” among the search results were retrieved for a full‐text review to assess inclusion criteria. The primary criterion for inclusion was the presence of individual‐level information on the prevalence of fungal infections in unmanipulated field settings. As relevant studies were scarce, we aimed to collect as comprehensive a sample of such papers as possible by applying a mixture of search methods, partly at the cost of compromising the repeatability of the search procedure. Nevertheless, as all our search procedures were strictly neutral with respect to the focal questions of our study (e.g., we did not use key words referring to high infection rates, like “epidemics” or “outbreak”), biases stemming from the search process are unlikely (see, however, Discussion for other sources of potential bias).

From each source paper deemed suitable, we extracted data on sample size (i.e., the number of insect individuals examined) and fungal prevalence (the share of individuals with signs of fungal infection) within the sample, along with the taxonomic identities of both insects and fungi. Additionally, we extracted information on geographical location, time of year, and, for herbivores, the growth form of the food plant, where available. To obtain a metric of fungal prevalence at the spatio‐temporal scale, we assigned a unique identifier to each documented insect‐fungus interaction at a particular study site (e.g., 15% individuals of insect species A infected by fungal species B at location Z and time T). Observations within 10 km and < 1 week apart were merged and treated as a single observation. Observations of zero EPF prevalence were disregarded unless they were merged with some non‐zero observations due to proximity in space and time (see above). All quantitative conclusions presented below should therefore be interpreted as applying only to cases where insect‐fungus interactions were confirmed to be present.

## 3 Data Analysis

**FIGURE 1:**
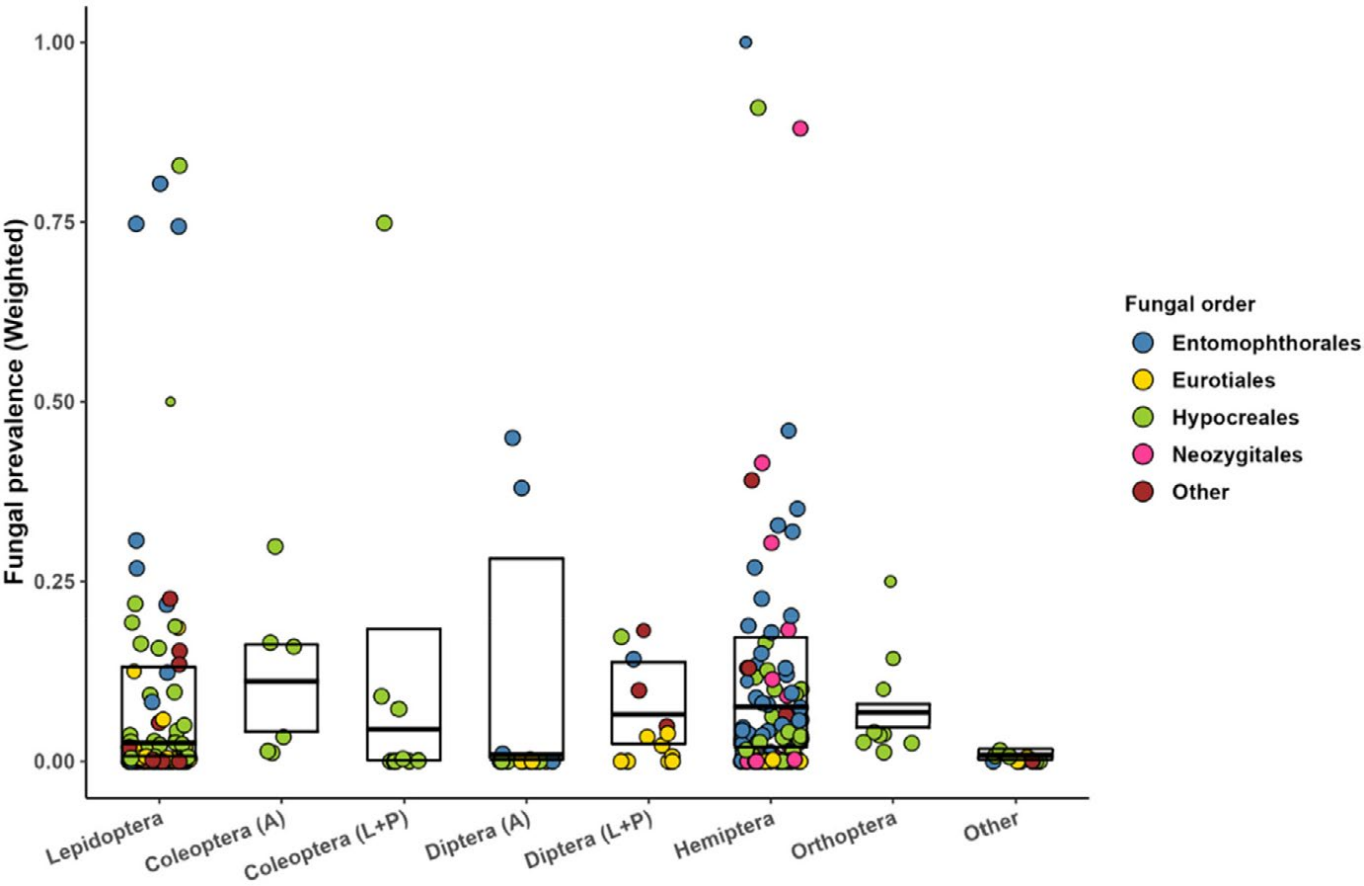
Prevalence of entomopathogenic fungi (all species pooled), presented separately for insect orders, and adult (A) vs. immature (larval and pupal; L+P) stages of those holometabolous orders for which such data were available. Boxplots represent the weighted median and quartiles of estimates of prevalence, which were derived from primary studies through a meta‐analytic approach. Each individual point represents a weighted mean prevalence of EPF for a single insect species, calculated across all observations within the same study (but not from different studies). To assign appropriate weights to the observations, we determined the standard error (SE) for each observation using the binomial formula $\sqrt{\frac{p\left(1-p\right)}{N}}$, applying *p* = 0.5 when *N* < 10 to mitigate small sample size concerns. Each observation was weighed by 1−2×SE, resulting in smaller weights for observations based on small and/or variable samples and near‐unit weights for observations based on large samples. The size of the points is proportional to the weight assigned.

**FIGURE 2:**
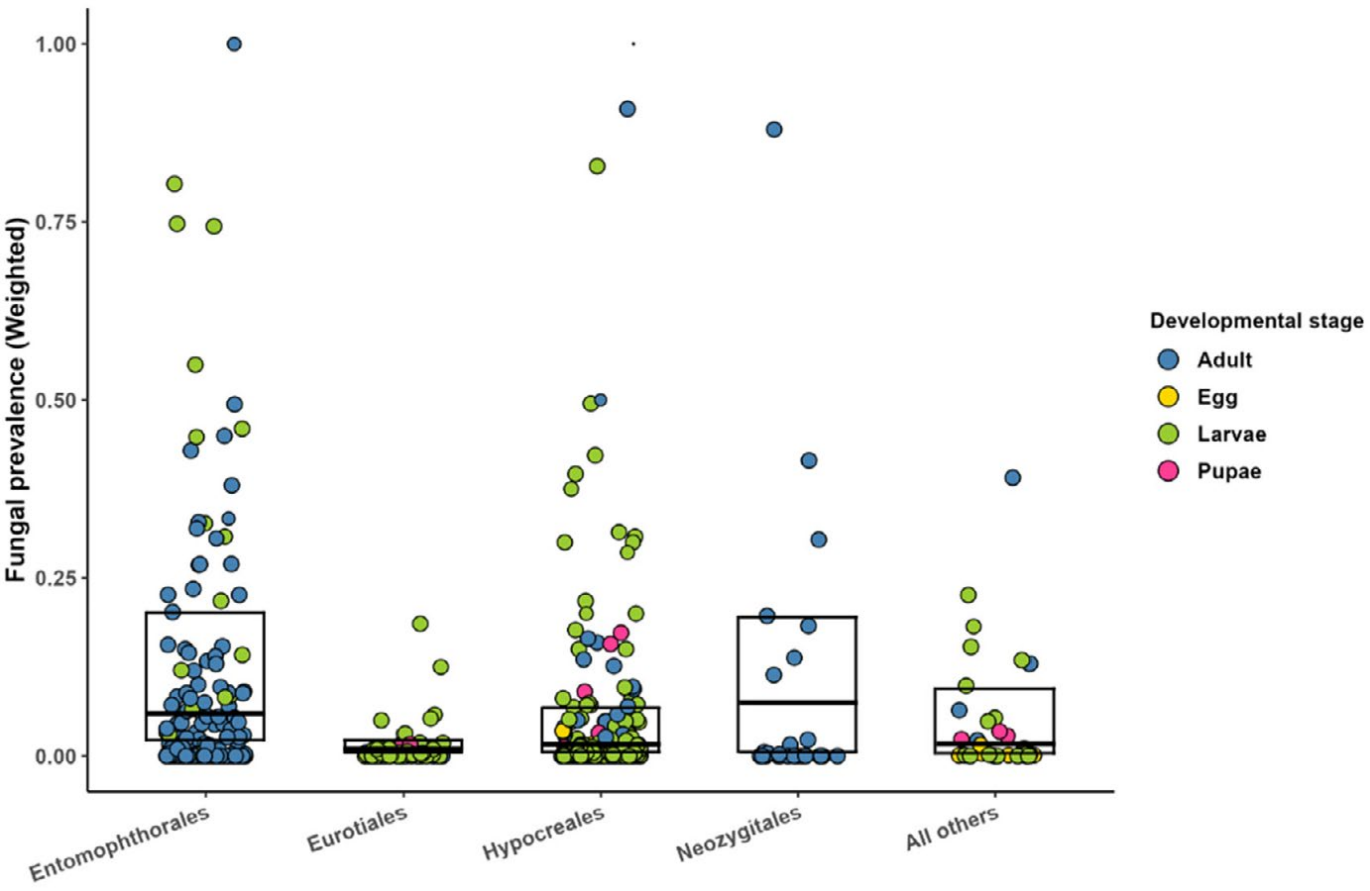
Observed prevalence of fungal infection, classified by fungal orders. Different colors refer to different developmental stages of insects. Dot size corresponds to the meta‐analytic weight of each observation (see Figure 1 for details).

**FIGURE 3:**
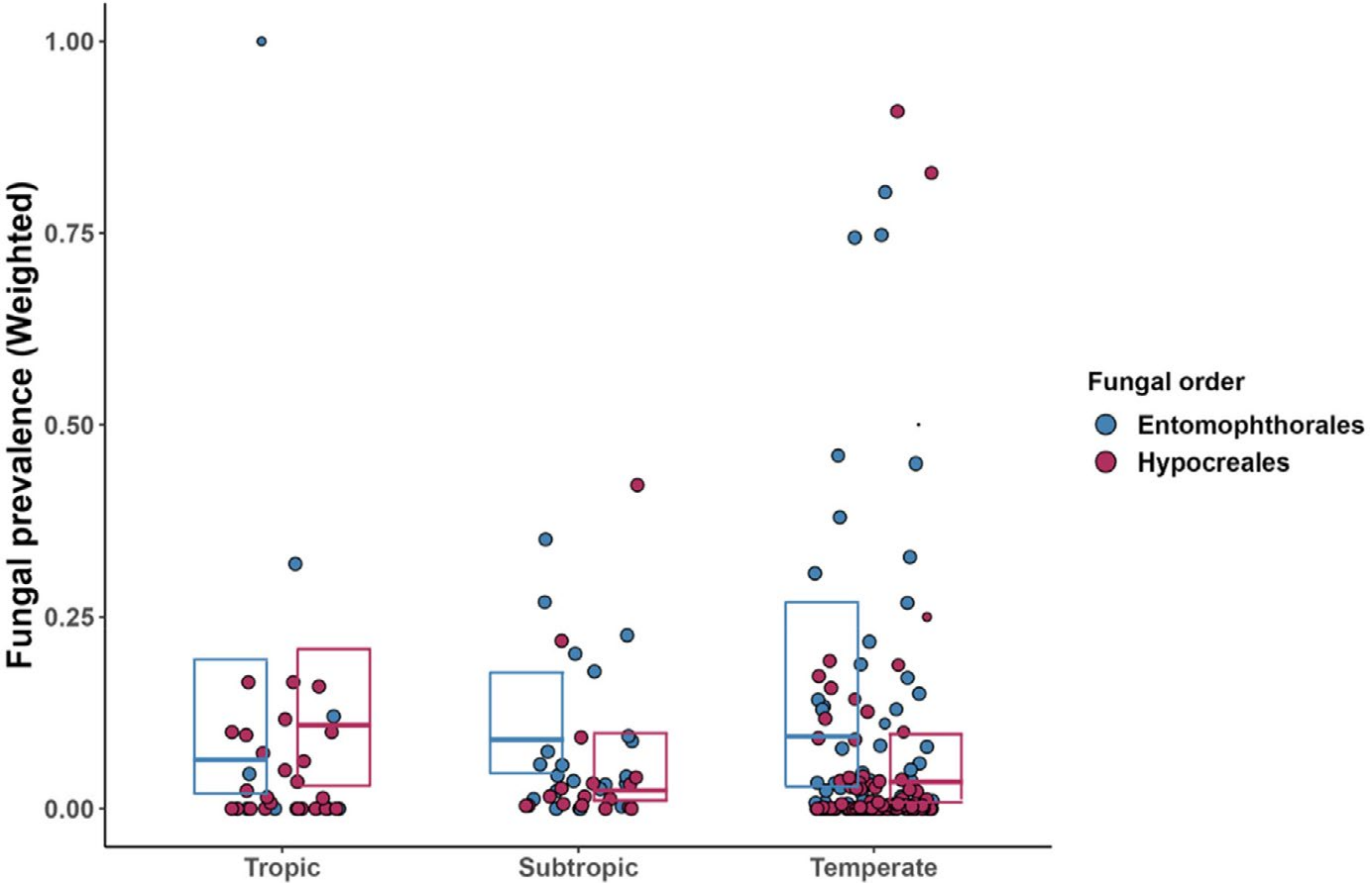
Prevalence of EPF infecting insects within three latitudinal zones: (tropics: 0°–23.43°, subtropics 23.43°–35°, and temperate 35°–65°). Dots show the observations for the two largest orders of entomopathogenic fungi (see Figure 1 for further details). Boxplots show the weighted medians and quartiles of the respective fungal orders.

**FIGURE 4:**
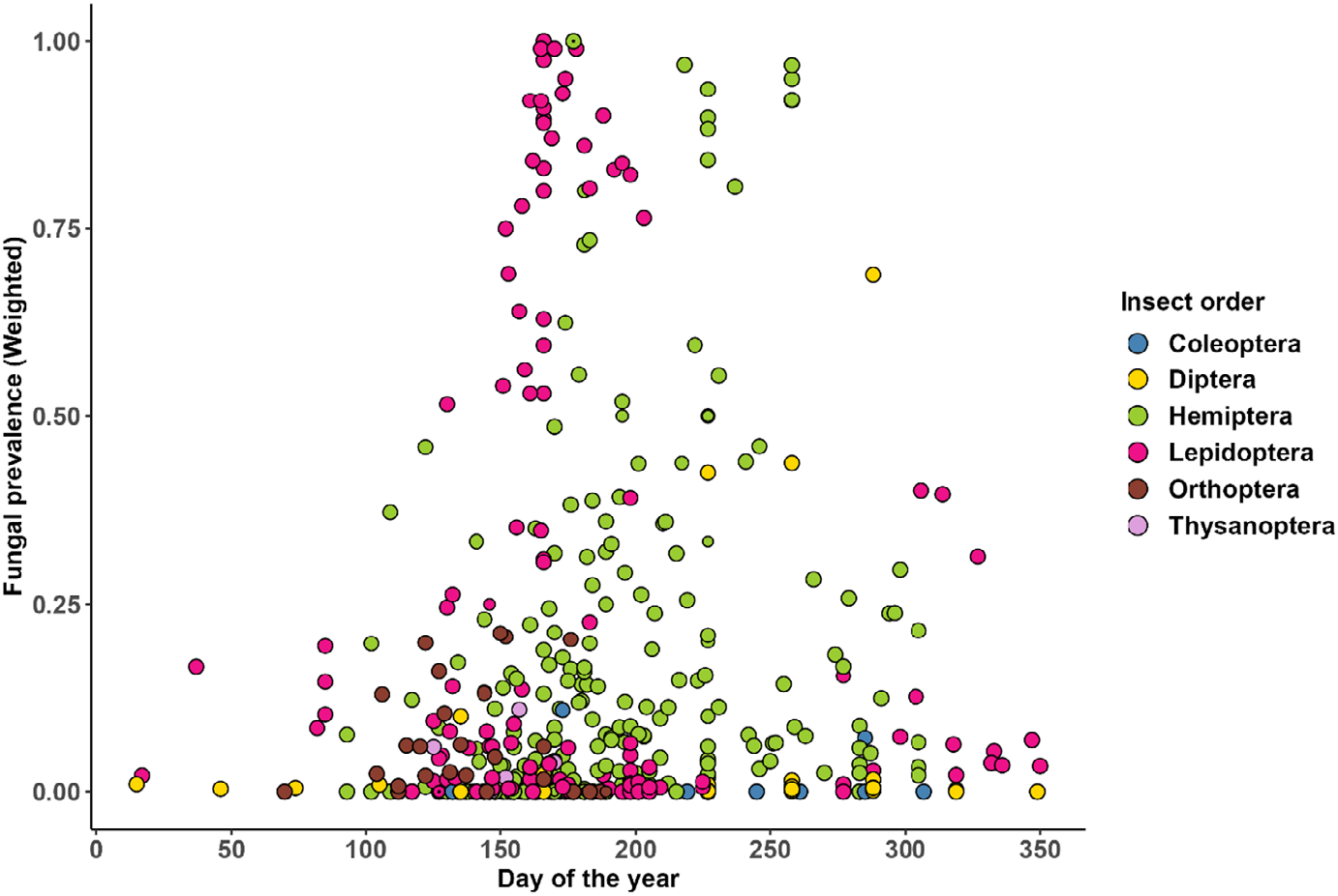
Dependence of fungal prevalence on calendar date in the temperate zone (latitude 35°–65°). Dot size corresponds to the meta‐analytic weight of each observation (see Figure 1 for details).

We first derived meta‐analytic descriptive statistics of EPF prevalence, organizing the observations in three different ways: by insect order (Figure 1), fungal order (Figure 2), and geographic region (based on latitude, Figure 3). We further tested for differences in fungal prevalence among (1) insect orders, (2) fungal orders, (3) geographic regions, and (4) growth forms of the host plants of the insects. This was done by fitting a binomial generalized linear mixed model (GLMM) with a likelihood ratio test to the aggregated data, incorporating source‐level and observation‐level random effects. The details of the statistical approach are provided in the legends of respective figures (Figures 1, 2, 3, 4).

Additionally, for the subset of data originating from the temperate zone (latitude 35°–65°) in the Northern Hemisphere, we examined the dependence of fungal prevalence on calendar date. We first applied a GLMM to the entire data set, with the second‐order polynomial effect of date as a fixed factor (see Appendix A for details of the analysis). Such an analysis combines two different phenomena. First, host insects characterized by different prevalences of EPF may not be evenly distributed throughout the vegetation period (e.g., heavily infested larvae of a moth may occur only in June). Second, fungal prevalence may display temporal trends within particular host–parasite systems (e.g., in a particular population of grasshoppers, there may be more infected insects in late compared to early summer). To isolate the latter phenomenon, i.e. system‐specific seasonal trends, we calculated the slope of logistic regression for each time series longer than 10 days, capturing the dependence of fungal prevalence on calendar date. We then tested whether the meta‐analytically weighted average of the slope values differed from zero and whether these slopes showed a dependence on calendar date (see Appendix A for statistical details).

## 4 Results

### 4.1 Data Set

We identified 79 papers reporting quantitative data on the prevalence of entomopathogenic fungi in field populations of insects. These sources provided 1273 records of insect‐fungus interactions (a particular insect species infected by a particular fungus, see Methods), involving a total of 104 insect species and 122 species of fungi. Just a minority of the studies (25) were based on populations studied in habitats that could be classified as natural (i.e., with minimal anthropogenic influence). The remaining estimates originated from agroecosystems of various types (fields, orchards), roadsides, and urban environments. This reflects the high share of such studies focusing on insects of agricultural importance, as evidenced by the high representation of the ‘pest insect orders’, such as Hemiptera (*n* = 614) and Lepidoptera (*n* = 433) in our dataset (Table 1). Among the other insect orders, only Coleoptera and Diptera were represented in notable numbers, with just single reports available for four further insect taxa (Blattodea, Hymenoptera, Orthoptera, and Thysanoptera).

**TABLE 1:** Observations of insect‐fungus interactions in natural populations by insect orders.

Insect order	No of studies	Populations studied	Fungal orders associated
Blattodea	1	1	Hypocreales
Coleoptera	8	69	Hypocreales
Diptera	9	104	Blastocladiales (6), Entomophthorales (49), Eurotiales (8), Hypocreales (41)
Hemiptera	33	726	Entomophthorales (481), Eurotiales (5), Hypocreales (97), Microascales (1), Neozygitales (112), Saccharomycetales (1), Other (29)^a^
Hymenoptera	1	2	Eurotiales (1), Hypocreales (1)
Lepidoptera	30	540	Capnodiales (3), Entomophthorales (118), Erysiphales (3), Eurotiales (44), Glomerellales (3), Hypocreales (323), Melanosporales (3), Microascales (3), Mortierellales (2), Mucorales (2), Pezizales (3), Pleosporales (5), Other (28)^a^
Orthoptera	1	46	Hypocreales
Thysanoptera	1	28	Capnodiales (1), Hypocreales (27)

^a^ “Other” means that the fungus was not identified or when data of different orders were pooled.

The EPF in our dataset belonged to 15 fungal orders across four phyla known to contain EPF. Members of Entomophthoromycota (order Entomophthorales) and Ascomycota (Hypocreales) clearly dominated among the observations (Table 1). Additionally, we found data on EPF belonging in one phylum not generally considered to harbor entomopathogenic fungi—Mortierellomycota (Naranjo‐Ortiz and Gabaldón 2019; Tedersoo et al. 2018).

### 4.2 Mean Prevalence

The weighted median prevalence of fungal entomopathogens varied from 0.8% to 11% across insect orders (split by development stages in some cases, Figure 1), with the highest values derived for adult Coleoptera and all Hemiptera, 11% and 6.9%, respectively (Figure 1). However, the differences in median prevalence among insect orders did not attain statistical significance. Just 82 interactions (5.4%, in 27 papers) could be classified as reaching epidemic level (mortality over 70%), while the vast majority of observations (*n* = 955) reported fungal prevalence of 10% or less. When the observations were classified by fungal order, the highest median prevalence was associated with Neozygitales (6.1%) and Entomophthorales (5.5%), followed by Hypocreales (1.4%) and Eurotiales (< 1%; Figure 2), the among‐order differences being statistically significant (χ^2^ = 28.53, df = 4, *p* < 0.001).

### 4.3 Differences in Time and Space

Overall, for the temperate data set, fungal prevalence showed some increase with calendar date within the growing season, with a peak at about midsummer (Figure 4). Both the positive linear trend (estimate = 2.66, SE = 0.03, *z* = 96.51, *p* < 0.001) and the negative quadratic term (estimate = −0.29, SE = 0.01, *z* = −22.4, *p* < 0.001) were statistically significant. When assessing whether fungal prevalence tends to increase or decrease with date within particular time series, the meta‐analytically weighted average of the corresponding slopes of the logistic curves (see Material and Methods and Appendix A) was slightly but significantly larger than zero (*b* = 0.0019, SE = 0.0009, *t* = 2.18, df = 13, *p* = 0.048) and showed a decrease with calendar date (*b* = −0.0014, SE = 0.0005, *t* = 2.91, df = 48, *p* = 0.006). The latter result confirms the non‐linear character of the relationship between fungal prevalence and date, indicating a tendency for more frequent decreasing trends in prevalence later in the season.

**FIGURE 5:**
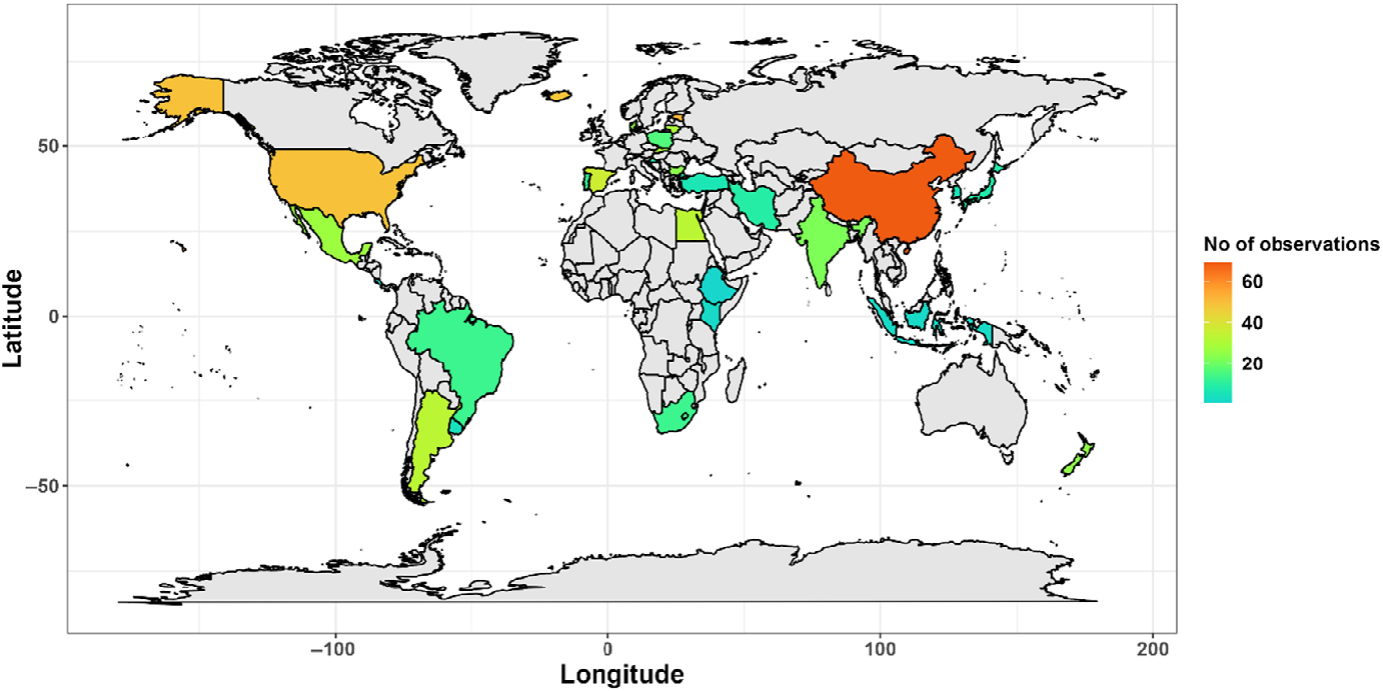
Geographic location of case studies on entomoptahogenic fungi used in the present review.

The primary case studies were clearly biased toward moderately high latitudes, mainly originating from China, the USA and Europe (Figure 5). When the data on EPF prevalence were divided into three climatic zones based on latitude (Figure 3), we revealed a (non‐significant) tendency for lower median prevalence at temperate latitudes (χ^2^ = 11.34, df = 7, *p* = 0.12). There was, however, a significant interaction between fungal taxon and latitude (χ^2^ = 13.84, df = 2, *p* < 0.0001; Figure 3), with the prevalence of Hypocreales increasing toward the equator, while that of Entomophthorales increasing toward the poles.

**FIGURE 6:**
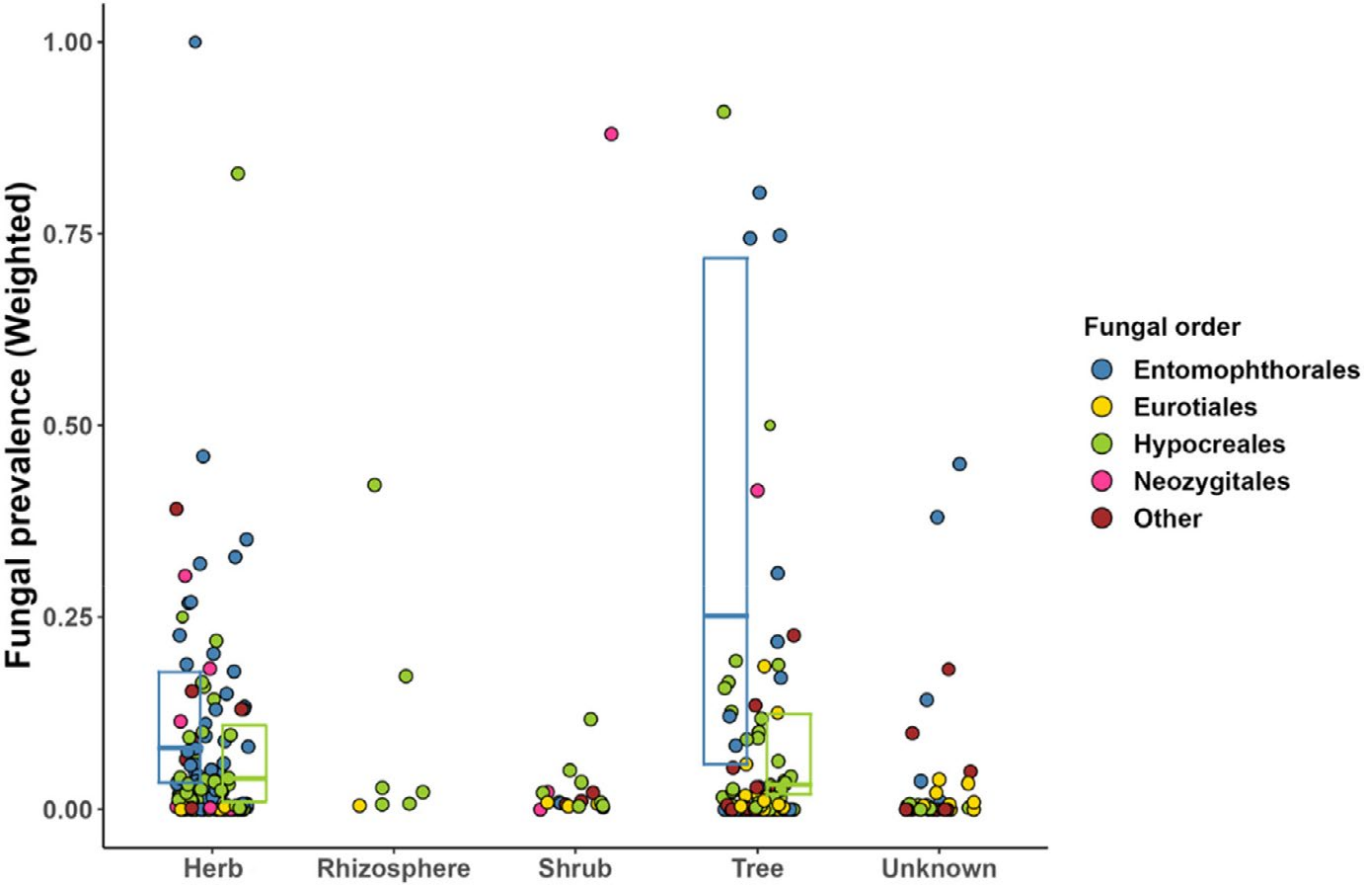
Fungal prevalence according to the plant growth form the host insect is associated with. Boxplots show the weighted medians and quartiles for the two largest orders of entomopathogenic fungi. Dot size corresponds to the meta‐analytic weight of each observation (see Figure 1 for details).

We further classified the observations by the type of host plants of the (herbivorous) insects (divided into herb, shrub, or tree, with the rhizosphere serving as the fourth class for soil‐dwelling insects; Figure 6). Sufficient data were available for a statistical analysis of the association between two growth forms (herb and tree) with the two main fungal orders (Hypocreales and Entomophthorales). The effect of plant growth form on fungal prevalence was clear (χ^2^ = 6.85, df = 1, *p* < 0.01), with higher prevalence on plants classified as trees. However, no interaction between the growth form and fungal taxon could be shown (χ^2^ = 0.22, df = 1, *p* = 0.64).

## 5 Discussion

### 5.1 The Current Knowledge

The number of studies reporting quantitative data on mortality caused by fungal pathogens in field populations of insects is far from impressive. The ecological understanding of EPF significantly lags behind our knowledge of insect mortality due to predators and parasitoids. Additionally, the available data are strongly skewed in terms of both insect and fungal taxa, as well as the ecological settings (biomes and habitats) in which their interactions have been observed. This must be largely because different systems vary in both their research appeal and the feasibility of studying them.

Hemiptera and Lepidoptera stand out with far more EPF records available than for any other insect order. This imbalance can be explained by the pest status of many representatives of these orders. Hemiptera, as sucking insects, are challenging to target with biocontrol agents other than fungi, which at least partly explains the extensive focus on them (Boomsma et al. 2014; Samson, Evans, and Latge 2013). Similarly, many lepidopterans are well‐known generalist pests of various crops, prompting research efforts to find efficient natural enemies (Hesketh et al. 2010; Lacey et al. 2015). Nevertheless, the disproportional representation of Lepidoptera in fungal infection records may also have biological grounds. Specifically, the various life stages of these insects often inhabit distinct environments, resulting in exposure to a range of different pathogens. For instance, because folivorous moth larvae typically pupate in the soil, lepidopterans encounter diverse assemblages of abundant facultative pathogens that persist in this stable environment (Roy and Cottrell 2013).

With respect to the representation of different fungal taxa, most papers to date have focused on just a few well‐known EPF species, especially those used in biocontrol. For some examples, consider *Entomophthora maimaiga* Humber, Shimazu, and Soper used in the USA against the gypsy moth ( *Lymantria dispar* Linnaeus), *Metarhizium anisopliae* (Metchnikoff) Sorokin in Brazil for managing spittlebugs, and *Beauveria bassiana* (Balsamo‐Crivelli) Vuillemin in China for corn borers (Hajek and Eilenberg 2018). Similarly, we may have some disposition to focus on clades well known to include entomopathogens, and to overlook the occasional incidence of this life style in other fungal taxa, such as Blastocladiomycota, Basidiobolomycota, Zoopagomycota, Kickxellomycota, Mortierellomycota, and Mucoromycota (Naranjo‐Ortiz and Gabaldón 2019). Likewise, lesser‐known entomopathogens within Ascomycota, such as species from the genera *Aspergillus, Fusarium*, and *Penicillium*, might be underrepresented among taxa recorded as pathogens of insects. This is because of their ability to colonize various dead tissues, which may have led to considering them saprotrophs. However, accumulating evidence suggests that such fungi could be common opportunistic pathogens infecting a wide range of insect taxa (Gielen et al. 2021; Seye et al. 2014; Sharma and Marques 2018).

Studies conducted in agroecosystems (54 papers) significantly outnumber those performed in more natural settings (25 papers), again reflecting a strong emphasis in EPF research on their biocontrol potential. Consistently, research on EPF infections has predominantly focused on insects that feed on economically significant crops, such as wheat, tobacco, and corn. As a result, a large portion of the relevant data comes from countries with extensive agricultural sectors (USA, China, and Argentina). Studies performed in natural ecosystems have mostly been conducted in temperate broad‐leaved and mixed forests (USA, Spain, Bulgaria, and Estonia), but they are still primarily economically motivated in the context of developing biocontrol measures against invasive species (Elkinton et al. 2019; Kovač et al. 2021; Reilly et al. 2014) or pests (Draganova et al. 2013; Nielsen et al. 2001).

### 5.2 Quantitative Conclusions

Our analysis suggests that overall mortality levels attributable to fungal infections in insect populations are generally low (below 10%), and consistently so across various insect and fungal taxa, as well as among different geographic regions. It is also important to note that the respective case studies are likely to preferentially focus on situations with a higher prevalence of EPF and higher population densities of the hosts, rendering our data set susceptible to publishing bias. There are thus reasons to believe that our meta‐analytically estimated medians, despite being already low, are still likely to be overestimates rather than underestimates. Therefore, the overall conclusion regarding the generally low prevalence (compared to mortality caused by predators and parasitoids) of EPF in insect populations appears to be robust.

Only 5.4% of observations, although 33% of papers, documented epidemic levels of fungal infections, defined here as 70% mortality and above. Some such examples involve the aphids *Schizaphis graminum* Rondani and *Sitobion avenae* Fabricius, infected with the entomophthoralean fungi *Pandora neoaphidis* (Remaud & Hennebert) Humber (83.9%, *n* = 205) and *Zoophthora radicans* (Brefeld) Batko (97.7%, *n* = 257), respectively, in the Argentinan pampas (Manfrino et al. 2014). High fungal prevalence has also been reported for the gypsy moth when infected by *Entomophthora maimaiga* (78%–99%, *n* = 100) in North America (Reilly et al. 2014). Epidemics are, however, not only limited to the fungal order Entomophthorales, as exemplified by the beetle *Alphitobius diaperinus* Panzer infected with the hypocrealean fungus *Beauveria bassiana* (72%–100%, *n* = 16–235) in Brazil (Alves et al. 2005). Nevertheless, reports of epidemic prevalence are nearly always based on small sample sizes and may thus represent highly local phenomena. The largest of such samples comes from a Japanese study on the aphid *Orosanga japonica* Melichar infected by *B. bassiana* (89.7%, *n* = 411, Akıner et al. 2020).

The low incidence of epidemic prevalence is in some contrast with the extensive laboratory research showing epidemic potential of EPF (Hossain, Rahman, and Khan 2017; Mora‐Aguilera, Cortez‐Madrigal, and Acevedo‐Sánchez 2017). Such studies, however, often focus on the physiological susceptibility of insects, which may not accurately reflect their ecological susceptibility (Hesketh et al. 2010). Indeed, laboratory‐based infections frequently appear unnatural, are conducted with high dosages and under favorable circumstances for the protagonist (Roy and Cottrell 2013). Also, most probably, epidemic situations are much more likely to be reported in research papers than cases of low or moderate prevalence. We thus feel confident to conclude—based on low absolute numbers of such reports and their small sample sizes—that epidemics caused by fungal pathogens are rare phenomena in the insect world.

Overall, even if data to directly support such conclusions in a general form are still scarce, there is no evidence conflicting with the emerging generalization (Gielen et al. 2021, 2023; Gielen, Põldmaa, and Tammaru 2022; Poitevin et al. 2018) that insect populations harbor species‐rich assemblages of pathogenic fungi, whereas infections only rarely reach epidemic levels.

### 5.3 Future Perspectives

#### 5.3.1 Systematic Sampling

Quite obviously, the empirical data currently available are insufficient for deriving reliable quantitative estimates of mortality attributable to fungal infections across different insect and fungal taxa, and different habitat types. To obtain an unbiased picture, we need studies on systems selected without prior knowledge about the (high) prevalence of fungal diseases (see Draganova et al. 2013; Gielen, Põldmaa, and Tammaru 2022; Poitevin et al. 2018 for some examples). A practical complication is that direct quantitative recording of mortality due to a fungal disease is feasible only when both infected and healthy insects can be readily counted, which is rarely the case. This assumption is met for insects with a sessile lifestyle, like apterous aphids (Barta and Cagáň 2003; Rhainds and Messing 2005) and coccids, or those with endophagous larvae (Gielen et al. 2023; Hyblerová, Medo, and Barta 2021). For many others, such as externally feeding lepidopteran larvae, a practical approach might be collecting mature larvae approaching pupation, and rearing them to adults in the laboratory. This approach, however, rests on the assumption that infection by fungal propagules occurs during the larval stage (which is likely in many systems, Boomsma et al. 2014; Elliot et al. 2000; Vega 2018), while the visual signs of fungal disease appear in the pupal stage.

#### 5.3.2 Targeting Entire Assemblages

Most field research on EPF has concentrated on tracking the occurrence of just a few well‐known species rather than documenting the entire assemblages of EPF associated with insect populations. This is unfortunate because recent empirical studies by the authors show that mortality imposed by fungal entomopathogens on insect populations results from the combined effects of many species, often from very distant branches of the phylogenetic tree (Gielen, Põldmaa, and Tammaru 2022; Gielen et al. 2022, 2023). Very few other studies have taken such assemblage‐oriented approach (Amatuzzi et al. 2017; Poitevin et al. 2018).

#### 5.3.3 Density Dependence

For pathogens to effectively regulate insect populations, they should operate in a density‐dependent manner, something of primary importance also for using these organisms in biological control (Hajek and Eilenberg 2018). Since, in temperate zones, EPF fruiting bodies primarily release spores in the summer after host infection, Bonsall (2004) suggested that EPF may exert a delayed density‐dependent effect on insect population dynamics. However, further research is needed as, to the best of our knowledge, density dependence mediated by EPF has only been demonstrated in one species of Lepidoptera, the beech caterpillar (*Syntypistis punctatella* Motschulsky) through time series analysis (Kamata 2000).

#### 5.3.4 Interactive Effects

For generalist fungi, such as those from the order Hypocreales, it has been hypothesized that the susceptibility of an insect host to infection should be strongly dependent on its physiological state (Boomsma et al. 2014; Takov et al. 2021). However, a study addressing this question did not confirm this hypothesis (Gielen, Põldmaa, and Tammaru 2022). If it existed, such a relationship would be of ecological significance by facilitating interactive effects: the nutritional stress of host insects could be amplified by increased mortality among the weakened larvae.

#### 5.3.5 The Effects of Host Plant

The plant that a herbivorous insect feeds on provides the template for the fungus‐insect interaction. Our meta‐analysis found an indication that the environment may indeed matter: rhizosphere‐associated insects were almost exclusively found to be infected with species from the order Hypocreales, contrasting with the much higher diversity of fungi in other environments (Figure 6). The role of the host plant acquires further significance in light of the so‐called “bodyguard hypothesis”, which suggests that plants may create favorable conditions for the pathogens of their herbivores, “allowing” them to grow as endophytes (Elliot et al. 2000). Although this hypothesis has received little attention from ecologists so far (see, however, Parsa, Ortiz, and Vega 2013; Vega 2018), there is evidence that getting infected with a particular fungus may indeed depend on the species of the host plant the insect larva is feeding on (Gielen, Põldmaa, and Tammaru 2022; Gielen et al. 2022). The affinity of certain pathogens for certain plant species or even growth forms could provide novel insights into the evolution of tritrophic interactions and may also reveal further ecological factors behind the evolution of host range in insects (Jaber and Ownley 2018; Vidal and Murphy 2018).

#### 5.3.6 The spatial ecology

The spatial ecology of insect‐fungus interactions remains another understudied aspect of EPF ecology. On the global scale, our study provided some indication of higher infection rates in the tropics compared to temperate areas. Nevertheless, the data are still clearly insufficient to conclusively confirm consistency of these results with similar trends in predator‐induced mortality (Roslin et al. 2017; Zvereva and Kozlov 2021). A key concern regarding such a geographical comparison arises from the differing proportions of applied studies in the pool of relevant case studies. In particular, case studies in the tropics tend to be conducted in applied contexts, often focusing on cases with high infection rates. At the other end of the spatial scale, patchiness in EPF infection risk may influence the spatiotemporal dynamics of insect populations, and create selective pressures on insect dispersal. Just a handful of surveyed articles are interpretable in this context (Fuentes‐Rodríguez et al. 2020; Gielen, Põldmaa, and Tammaru 2022), with the data yet clearly insufficient for any generalizations.

#### 5.3.7 Seasonal Patterns

It is intuitive to expect that, in temperate regions, the incidence of fungal infections should increase toward the end of growing season. Indeed, EPF are expected to propagate throughout the season, and as population densities of their (multivoltine) hosts may increase, conditions for the infections to spread should improve. Moreover, the quality of resources for herbivorous insects typically decreases toward the end of the season (Feeny 1970; Tikkanen and Lyytikäinen‐Saarenmaa 2002; Roslin and Salminen 2009), making the insects potentially more susceptible for infections. The results of the present review are broadly consistent with these expectations, although the relationship appears not to be linear (Figure 4). Studies specifically focusing on this question are clearly needed.

#### 5.3.8 Biology of the Fungi

Taxon‐specific biological traits of different EPF likely play an important role in shaping their interactions with insects and deserve further research. This is here exemplified by the latitudinal differences in the taxonomic composition of EPF (Figure 3): prevalence of Hypocreales tends to be higher in the tropics, while Entomophthorales are more common in temperate areas. Currently, we could only speculate about the reasons for this pattern. Similarly, we see no straightforward explanation for the tendency of higher EPF prevalences on trees compared to other plants (Figure 6).

#### 5.3.9 Host Specialization

While species of Entomophthorales are known as host specialists, hypocrealean fungi have traditionally been considered less host‐specific (Samson, Evans, and Latge 2013; Vega and Kaya 2012). However, research in this direction is aggravated by problems in fungal species delimitation. Recent molecular studies have revealed a previously unrecognized diversity of cryptic species within various well‐known fungi (like *Beauveria bassiana* and *Metarhizium anisopliae*; Bonsall 2004; Hesketh et al. 2010), complicating the establishment of host ranges based on older data. Studies from recent decades applying molecular phylogenetics have revealed rather extreme specialization among these newly recognized species (Sung et al. 2007; Vega et al. 2012; Wraight, Inglis, and Goettel 2007). A clearer understanding of host specificity in EPF—particularly regarding the extent of natural enemy sharing among insect species—remains essential for elucidating their roles in population dynamics within natural insect communities.

## Author Contributions

**Robin Gielen:** conceptualization (equal), data curation (lead), formal analysis (equal), methodology (equal), visualization (lead), writing – original draft (equal). **Kadri Ude:** data curation (lead), formal analysis (supporting), visualization (equal), writing – review and editing (supporting). **Ants Kaasik:** data curation (supporting), formal analysis (equal), writing – review and editing (supporting). **Kadri Põldmaa:** conceptualization (equal), supervision (equal), writing – review and editing (supporting). **Tiit Teder:** conceptualization (equal), formal analysis (equal), methodology (equal), writing – review and editing (supporting). **Toomas Tammaru:** conceptualization (lead), formal analysis (supporting), funding acquisition (lead), methodology (equal), project administration (lead), writing – original draft (equal).

## Conflicts of Interest

The authors declare no conflicts of interest.

## Data Availability

Data and codes are accessible at https://doi.org/10.15156/BIO/2959355.

## Appendix A

### Statistical Details of the Analysis of the Relationship Between Calendar Date and Infection Rate

For subset of the data originating from the temperate zone (latitude > 35°), we studied the dependence of infection rate on calendar date. First, we applied a GLMM on the entire data set with the second‐order polynomial effect of date as fixed factor. The date was centered and scaled to unit variance for numerical stability of the fitting process. The random factor structure included research paper the data were extracted from, year within paper and spatial cluster within paper and year. Such an analysis combines the effects of phenological distribution of host–parasite systems with different infection rates, and the phenology‐related changes within such systems. To isolate the latter effect, for each time series longer than 10 days, we expressed the dependence in the infection rate on calendar date as the slope of logistic regression (b), the slope describing the direction and rate of the change of prevalence of EPF with season progressing. Time series longer than 1 months were divided into sections considered as separate time series: each slice of the series had to be no longer than 30 days in timespan and the endpoint of the previous slice was used as the starting point of next slice. Slices with < 3 observations were discarded as logistic regression parameter standard errors would not have been available for these slices. Mid‐point of each slice was used as the date value in the meta‐analysis. It was asked whether the meta‐analytically weighed average of the b‐values differs from zero, and whether the *b*‐values show a dependence on (scaled) calendar date. Random effect structure again included paper, year within paper and spatial cluster within paper and year. All statistical tests for the meta‐analyses performed were carried out with settings test = “t” and df = “contain” for improved approximation of the degrees of freedom of the t‐distributions.
